# Interactions of Co, Cu, and non-metal phthalocyanines with external structures of SARS-CoV-2 using docking and molecular dynamics

**DOI:** 10.1038/s41598-022-07396-w

**Published:** 2022-02-28

**Authors:** Wilson Luna Machado Alencar, Tiago da Silva Arouche, Abel Ferreira Gomes Neto, Teodorico de Castro Ramalho, Raul Nunes de Carvalho Júnior, Antonio Maia de Jesus Chaves Neto

**Affiliations:** 1 Laboratory of Preparation and Computation of Nanomaterials (LPCN), Federal University of Pará, C. P. 479, Belem, PA 66075-110 Brazil; 2 Pos-Graduation Program in Engineering of Natural Resources of the Amazon, ITEC, Federal University of Pará, C. P. 2626, Belém, PA 66050-540 Brazil; 3 Pos-Graduation Program in Chemical Engineering, ITEC, Federal University of Pará, C. P. 479, Belém, PA 66075-900 Brazil; 4 National Professional Master’s in Physics Teaching, Federal University of Pará, C. P. 479, Belém, PA 66075-110 Brazil; 5 Federal Institute of Pará (IFPA), C. P. BR 316, Km 61, Castanhal, PA 68740-970 Brazil; 6 Chemistry Department, Federal University of Lavras (UFLA), C. P. 3037, Lavras, MG 37200-000 Brazil

**Keywords:** Computational models, Computational neuroscience, Computational platforms and environments, Protein analysis, Protein function predictions, Software, Virtual drug screening

## Abstract

The new coronavirus, SARS-CoV-2, caused the COVID-19 pandemic, characterized by its high rate of contamination, propagation capacity, and lethality rate. In this work, we approach the use of phthalocyanines as an inhibitor of SARS-CoV-2, as they present several interactive properties of the phthalocyanines (Pc) of Cobalt (CoPc), Copper (CuPc) and without a metal group (NoPc) can interact with SARS-CoV-2, showing potential be used as filtering by adsorption on paints on walls, masks, clothes, and air conditioning filters. Molecular modeling techniques through Molecular Docking and Molecular Dynamics were used, where the target was the external structures of the virus, but specifically the envelope protein, main protease, and Spike glycoprotein proteases. Using the g_MM-GBSA module and with it, the molecular docking studies show that the ligands have interaction characteristics capable of adsorbing the structures. Molecular dynamics provided information on the root-mean-square deviation of the atomic positions provided values between 1 and 2.5. The generalized Born implicit solvation model, Gibbs free energy, and solvent accessible surface area approach were used. Among the results obtained through molecular dynamics, it was noticed that interactions occur since Pc could bind to residues of the active site of macromolecules, demonstrating good interactions; in particular with CoPc. Molecular couplings and free energy showed that S-gly active site residues interacted strongly with phthalocyanines with values ​​of − 182.443 kJ/mol (CoPc), 158.954 kJ/mol (CuPc), and − 129.963 kJ/mol (NoPc). The interactions of Pc's with SARS-CoV-2 may predict some promising candidates for antagonists to the virus, which if confirmed through experimental approaches, may contribute to resolving the global crisis of the COVID-19 pandemic.

## Introduction

At the end of 2019, SARS-CoV-2^1,2^, a new coronavirus, was recognized, causing a severe acute respiratory syndrome^3^, and its outbreak caused a global COVID-19 pandemic^4,5^ causing great concern to the population because of the high contamination rate, propagation capacity and lethality rate^6,7^. Blocks^8^ and restrictions^9^ were imposed to prevent the spread of COVID-19, as the control of an infectious disease is based on knowledge of its mode of transmission^10^. In this situation, the best ways to prevent the transmission of COVID-19 are through measures of social distancing^11,12^, reducing close contact between individuals^13,14^. But when social distance is not possible, some personal hygiene measures must be maintained^15^, as well as the use of masks^16,17^. With the progress of the COVID-19 pandemic, we can highlight the importance of materials science, in the search for new tools and technologies for antiviral research and treatment development^18^. Among the new tools to treat patients with COVID-19 is the development of a phthalocyanine-derived mouthwash that appears as a promising alternative for reducing the viral load of SARS-CoV-2 and the clinical improvement of infected patients who presented moderate symptoms^19,20^. Phthalocyanines (Pc) are chemical compounds with applications in various technological systems, such as pigments^21^, catalysts^22–24^, biosensors^25,26^, chemical sensors^27^, dyes^28^, and photodynamic therapy (PDT)^29–32^, because of its various electronic and optical properties^33^.

Arıcı and his co-authors (2013)^34^ synthesized and investigated some electrochemical and spectrochemical properties of metal phthalocyanines (MPc). The authors found that CuPc and CoPc perform better electron transfer reactions than NoPc, showing an improvement in the REDOX behavior of Pc aromatic rings. Thus, it can be understood that the addition of transition metals in the center of Pc provides the passage of electric current in its molecular structure. This increase in REDOX behavior in molecules is very important for several applications, especially for electrocatalysis and electro sensors. Using Pc as photosensitizing dyes in PDT^35^ is intended for treatments: antimicrobials^36,37^, antivirals^38,39^, actinic keratosis, Bowen's disease, skin cancer, or mycosis fungoid in stage I or II^40^. The study of the application of Pc as a viral inactivator has shown excellent results when tested on bovine viral diarrhea viruses (BVDV), influenza A virus (H3N2), poliovirus type 1 (PV-1), and human adenovirus type 5 (HadV5)^41^, immunodeficiency virus type 1 (HIV-1), HIV-2 and simian immunodeficiency virus strains in various cell types^42^. Some works with a computational approach show that Pc, when associated with other compounds^43^, can simultaneously bind to 3C-like protease (3CLpro), papain-like protease (PLpro), RNA-dependent RNA polymerase (RdRp), and the spike protein (S), which can serve as multi-target drugs; besides the high sensitivity of SARS-CoV-2 to photodynamic inactivation by water-soluble Pc (Zn-PcChol 8+) to eradicate pathogens in localized lesions, infected liquid media and various surfaces^44^. Pc can be used as filtering by adsorption on paints on walls, masks, clothes, air conditioning filters^45^.

**Figure 1:**
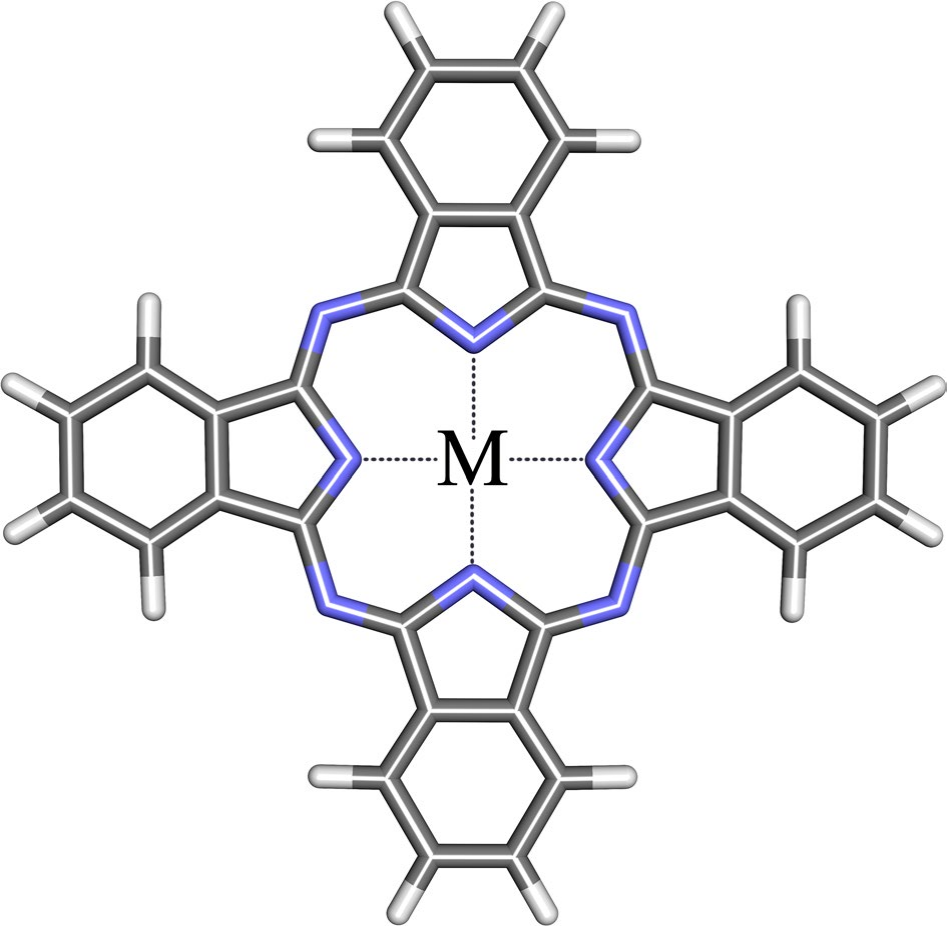
Phthalocyanine structure, M = Co, Cu e Non-Metal.

**Figure 2:**
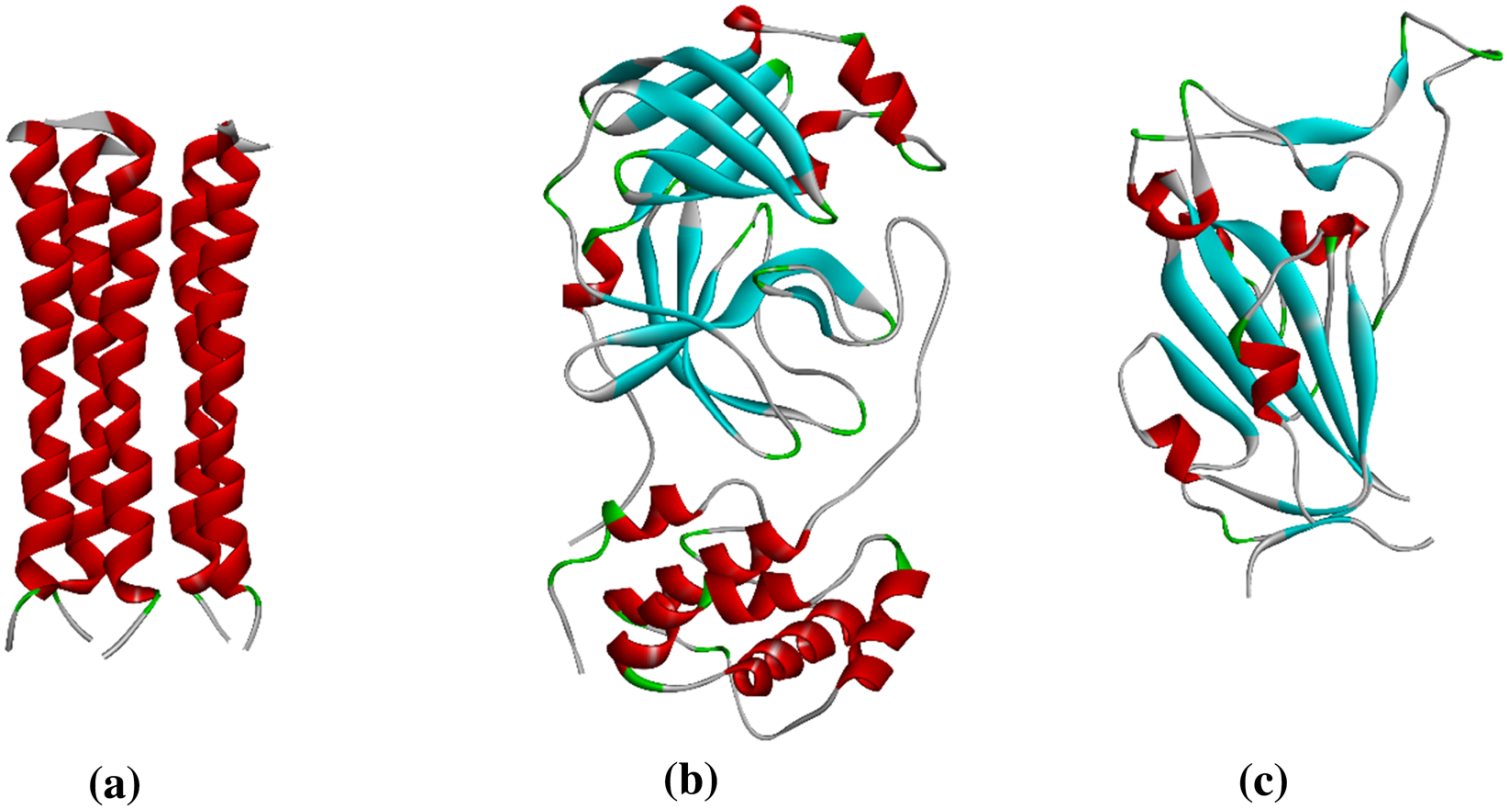
Macrostructures used: (**a**) E-pro; (**b**) M-pro (**c**) S-gly (Binding site).

In all biological processes, interactions between biomolecules play a fundamental role, as they constitute regulatory and metabolic networks, which are basic requirements for life. Molecular modeling techniques aim to monitor and analyze these interactions and also predict interactions or unknown structures of interacting biomolecules^46^. Among these techniques, docking stands out as a very important tool to predict the affinity of one molecule for another, a biomacromolecule^42,47^. In the present study, we analyzed the interaction of Cobalt (CoPc), Copper (CuPc), and non-metallic (NoPc) Pc (Fig. 1) with the surface structures of SARS-CoV-2 (Fig. 2); we used Molegro Virtual Docker 4.2 (MVD)^48,49^, which presented satisfactory results, as its scoring function can recognize metals^48,50^. The Molegro scoring function^51^ applies to all heavy atoms in the ligand and protein, including the cofactor atoms^52,53^. Although DOC simulations are very useful tools, they lack information about the dynamics of biomolecules and ligand complexes. For this step, we used the GROMACS 2020.2 software^54–56^, for the molecular dynamics (MD) simulations^57,58^ which could provide information on the deviation of the root mean square of atomic positions (RMSD)^59,60^, calculations using the generalized Born implicit solvation (GB) model^61,62^ and Gibbs free energy (G)^63,64^ and accessible surface area (SASA)^65,66^. Therefore, it is imperative that molecular docking, combined with other computational techniques provided reliable results. When performing MD simulations, the dynamic behavior of arrays^67^ can be monitored and probed at different time scales, allowing studies of fast internal motions and slow conformational changes for complex processes such as ligand binding to an active site or bending of protein^68,69^. The number of MD applications in medicines is always increasing and it would be almost impossible to name them all. When used together, the in silico and experimental procedures provide insight into the elaborate features of intermolecular recognition, making such a procedure good practice in discovering virus-inhibiting agents^70,71^. Thus, we intend to explore the interactions of Pc with the external structures of SARS-CoV-2 through a virtual search in an attempt to analyze an inhibition by or inactivation of the virus.

## Material and method

### Target preparation

All simulations were performed based on the receptor + ligand model^72^, where three structures were selected E-pro (PDB ID: 7K3G), M-pro (PDB ID: 6LU7), and S-gly (PDB ID: 7BZ5) from the Protein Data Bank repository (PDB; (www.rcsb.org/pdb)^73^. The receivers were optimized using Chimera 1.15.6 (CHM) software^74^ to find the optimal conditions that satisfied the various predefined targets, such additional complexity arises for tasks involving experimentation or computational calculations^75–78^ We use the minimized structure–function of the CHM, to clean up small molecule structures and improve localized interactions in larger systems. steep to first to alleviate highly unfavorable confrontations, followed by minimization of the conjugate gradient, which is much slower but more effective in achieving minimum energy after alleviating severe shocks. Potentially ambiguous or rare protonation states (displaced pKa), especially at binding sites and non-standard residues, should be checked and corrected before loads are assigned. For example, extra hydrogens can be excluded and atom types can be edited (before hydrogen addition). We use the default values ​​for the steep descent steps and standard conjugate gradient as a general-purpose scalar realization function for optimization Multi-target, where rankings are the limiting factor and its performance has been well-established using different single-purpose optimization algorithms, this allows a wide class of optimization algorithms to find quickly. Under ideal conditions, in this sense, we use the standard AMBERFF14SB^79^ force field to obtain the structure with the best conformation based on the set, where we also calculate its restricted electrostatic potential charges (RESP)^80,81^.

### Ligand preparation

Ligands were selected based on their stability regarding the software used. The 3D structures of the ligands were built in GaussView 6.0^82^ and the most stable conformer of each ligand was searched^83^ using the density functional theory (DFT) method^84^. The best conformer was chosen and optimized by the density functional theory (DFT) approach with the B3LYP /LANL2DZ method that includes dispersion interactions^85^ in Gaussian09 software. Thus, with LANL2DZ base sets, they have been widely used in quantum chemistry, particularly in the study of compounds or clusters containing heavy elements. These basic functions were obtained by the procedure of fitting pseudo-orbitals with Gaussian functions. LANL2DZ base sets are routinely employed also in density functional DFT calculations^86^. The optimized structures of all phthalocyanines were saved in pdb file format. All metals coordinated to Pc in this study were in the 2 + state^87^. The valence pseudo-orbitals were rehired using B3LYP hybrid exchange–correlation potentials. While the exponents of the primitive Gaussian functions and the contraction scheme were kept fixed at the original optimization level, the contraction coefficients were optimized through a step-by-step procedure that employs an SCF optimization for each contraction, building an input estimate for the Gaussian09.

### Docking protocol used in Molegro Virtual Docker

All necessary parameters have been specified using the Molegro Docking Wizard^88^. The search space was specified; a radius value is needed in the Molegro, which has been defined as 30 Å, to include all segments of the macrostructures in the search space. "MolDock SE" was selected for the search algorithm because of the number of rotating bonds and the magnitude of the ligands. MolDock uses a search algorithm that couples the cavity prediction algorithm to the differential evolution method^89^. This hybrid algorithm is known as guided differential evolution. The population size and the maximum number of iterations (minimization in each step of the MolDock SE algorithm) were fixed at 150 and 2500, respectively^90^. The number of poses was set to thirty, where independent instantaneous runs were performed. The main superiority of the Molegro is the completion of metal recognition^91^. Therefore, the use of Molegro is considered to provide more reliable results against binders containing different metals. Since the docking scores obtained from the Molegro do not refer to energy values, a transformation to binding constants was not performed. The scores were relatively evaluated. The scoring function used by MolDock Score^92^ derives from the PLP scoring functions, such that the scoring energy (Score) is defined by the sum of the energy terms of ligand–protein interaction (E_inter_) with the internal energy of the ligand (E_intra_)

1 $${E}_{score}={E}_{inter}+{E}_{intra}$$

In this scoring function, the equation has been optimized to improve scoring with a new term for hydrogen bonds and a new scheme for charges. Thus, the energy-related to the ligand–protein interaction (E_inter_) is defined as:

2 $${E}_{inter}=\sum_{i}\sum_{j}\left[{E}_{PLP}\left({r}_{ij}\right)+332.0\frac{{q}_{i}{q}_{j}}{4{r}_{ij}^{2}}\right]$$

The term E_PLP_ is a piecewise linear potential that uses two parameters, one to approximate the steric term (van der Waals) between atoms and a second for hydrogen bonds, this being a stronger potential. The second term describes the electrostatic interactions between charged atoms, a Coulomb potential where the distance-dependent dielectric constant is defined by D(r) = 4r. Multiplication by the numeral 332.0 transforms the electrostatic energy units to kcal/mol. To ensure that the contribution (electron-core attraction) is not greater than the approximation penalty (core-core repulsion), the electrostatic energy is disregarded at distances smaller than 2.0 Å.

The internal energy of the ligand (E_intra_) is the sum of the pairs of atoms in the ligand, excluding pairs of atoms connected by two bonds or less:

3 $${E}_{intra}=\sum_{i}\sum_{j}{E}_{PLP}\left({r}_{ij}\right)+\sum_{flexiblebonds}A\left[1-cos\left(m\theta -{\theta }_{0}\right)\right]+{E}_{clash}$$

where θ – represents the torsional angle of the connection.

The second term refers to the torsional energy, being parameterized according to the types of hybridization of the bonded atoms. If many torsions have been determined, the average contribution of the torsional energies of the bond is used. The third term (E_clash_), is related to the spatial issue of heavy atoms, assigning a penalty of 1000 if the distance between two of these atoms is less than 2.0 Â, and 10,000 if the heavy atom is outside the interaction site (delimited by the spherical grid). After the software predicts one or more promising poses, a series of additional energy terms are calculated at the end of the run. These terms are linearly combined, generating the "rerank score”.

### Electrostatic surface potential method

Electrostatic potential maps allow visualizing the charge distributions of molecules and the charge-related properties of molecules^110^. It also allows us to visualize the size and shape of molecules. In organic chemistry, electrostatic potential maps are invaluable in predicting the behavior of complex molecules^111^. The first step involved in creating an electrostatic potential map is to collect a very specific type of data: electrostatic potential energy^112^. We use Gaussian 09 to calculate the electrostatic potential energy at a distance from the molecule's nuclei^113^. Electrostatic potential energy is a measure of the strength of charges, nuclei, and nearby electrons in a particular position. To accurately analyze the charge distribution of a molecule, an enormous amount of electrostatic potential energy values ​​must be calculated. The best way to convey this data is to represent it visually, such as an electrostatic potential map. Data is calculated in an electron density model of the molecule derived from the Schrödinger equation^114,115^. To make the electrostatic potential energy data easy to interpret, a color spectrum, with red as the lowest electrostatic potential energy value and blue as the highest, is employed to convey the varying intensities of the electrostatic potential energy values^116^. Molecular electrostatic potential maps also illustrate information about the charge distribution of a molecule^117^. Electrostatic potential maps convey information about the charge distribution of a molecule because of the properties of the nucleus and the nature of electrostatic potential energy^118^. Thus, a high electrostatic potential shows the relative absence of electrons and a low electrostatic potential shows an abundance of electrons. This property of electrostatic potentials can also be extrapolated to molecules. Conformational analysis showed that the space accessed by these compounds was very different. The best pose from the molecular docking study was selected to generate the electrostatic potential maps.

### Protocol for MD simulations

MD simulations of Pc linked to SARS-CoV-2 surface macromolecules were performed using the CHARM36 force field^119^ as implemented in GROMACS version 2021.2^120^ in an explicit aqueous solution. The box was filled with single-point charge water molecules (SPC)^121^. Sodium and chloride ions were also added to the system. The nested positions provided by Molegro were used as initial structures and placed in a cubic box with dimensions 3.2 Å, 2.8 Å, and 2.2 Å, for E-pro, S-gly, and M-pro. The initial structures were later minimized in terms of energy with a steeper descent method^122^. The results of this minimization produced the initial structures for the MD simulations. Each system contained an average of about 12,200 atoms in total. The MD simulations were then performed with a constant number of particles, pressure, and temperature, NPT setting. The SETTLE algorithm^123^ was used to constrain the bond length and angle of the water molecules. Long-range electrostatic interactions were calculated using the Particle-Mesh-Ewald (PME) method^124^. A constant pressure of 1 bar was applied, water molecules and ions were separately coupled to a bath at 303 K with a coupling constant of 0.1 fs^125^ The equation of motion was integrated every 2 steps of time fs^126^. Each simulation was run for 100 ns and the systems were balanced for the first 10 ns. The analysis of the simulated trajectories and structures was carried out with the built-in tools of the GROMACS program. The PME method was applied to calculate the electrostatic interactions and the conjugate gradient algorithm^127,128^ was used to constrain covalent bonds involving hydrogen. Using the CPPTRAJ tool^129^, it was possible to extract the necessary information for the creation of Root Mean Square Deviation (RMSD) graphs^130,131^ and free energy tables^132^, all as a function of time. RMSD shows how much the protein structure changes during a simulation, the initial structure is usually crystallographic.

Through the TRJCONV module^133^, the necessary information for the creation of graphs of RMSD values ​​as a function of time was extracted. These values ​​indicate the deviations of the structures generated during the simulation about the initial structure obtained through molecular anchoring, that is, the stability and equilibrium of the system considering the dimension of time. Free energy defines the binding affinities of protein–protein and protein–ligand interactions, and the efficiency of possible binding also quantifies many other important processes, such as enzymatic reactions, electron transfer^134^, ion transport across membranes^135^, and solvation of small molecules^136^. We use the MM / PBGBSA method scripts^137,138^ to automatically perform all the necessary steps to estimate the free energy of the complex binding using these methods. However, it is generally approximated that no significant conformational change occurs after connection, so snapshots of the three species can be obtained from a single trajectory^139^.

### SASA method

SASA is calculated from MD trajectories^93^. It includes the hydrophobic, hydrophilic, and total solvent accessible surface area of ​​the protein molecule. The calculated surface area is the canonical surface area^94^. The extent to which amino acids interact with the solvent and the protein core is proportional to the surface area exposed to the solvent. Two solvation models can perform MD simulations of solvated systems: the explicit solvation model and the implicit solvation model^95^. Empirical methods, such as the SASA^96,97^, often provide simple and fast ways to assess solvation energy with an accuracy comparable to theoretical models. In the SASA approach, the free energy of solute solvation is expressed as the sum of the atomic contributions, weighted by their areas exposed to the solvent^98,99^. The continuous solvation model has stood out to describe electrostatic solvation. In this approach, the solute is considered as a cavity embedded in a dielectric medium. The corresponding free energy of electrostatic solvation can be accurately calculated using the solutions of the Poisson- Boltzmann Equation (BP)^100^, or approximately by using the generalized Born model (GB)^101^. In studies of the association of biological macromolecules with ligands, the generalized Born (GB) implicit solvation model is the most applied because of its lower computational cost^102,103^. The GB/SA combination has been recognized as an excellent choice for the implicit solvation treatment in biomolecular simulations^104^. Several optimizations of implicit solvation methods have been performed^105,106^ and are implemented in several packages of molecular modeling programs such as CHARMM36^107^, AMBER^108^, and XPLOR^109^.

## Results and discussion

### Results from Molegro

To evaluate docking results, it is conventional to use the best docking score. On the other hand, we use the Boltzmann weighted average of binding energies to obtain a more realistic result, since states with lower energy will occur more likely than those with higher energy in a system. We propose that such an approach is more rational concerning using the standard mean of the binding scores or selecting the best binding score. The data in Table 1 indicate that the absence of metal reduces the possibility of binding when compared to Pc's with metal. CoPc presents a greater possibility of interaction between CuPc and NoPc, for the three proteins considered in this study. This result may be related to the significant differences in the hybridization of the 3d states of Co and Cu transition elements with the states in the valence and conduction bands, as observed in the study of KLYSKO and SYROTYUK (2021)^140^. To assess docking results, it is conventional to use the lowest docking score which can create a better binding between the ligand and the protein. In this work, the lowest energy occurs between the M-pro protein with the CoPc and CuPc ligands, presenting a score of − 205.899 kcal/mol and − 202.862 kcal/mol, respectively.

**Table 1:** MolDock score values.

Complexes		MolDock Score (kcal/mol)
E-pro	CoPc	− 129.007
	CuPc	− 125.744
	NoPc	− 102.827
M-pro	CoPc	− 205.899
	CuPc	− 202.862
	NoPc	− 177.111
S-gly	CoPc	− 142.843
	CuPc	− 137.411
	NoPc	− 118.795

The MVD automatically identifies potential binding sites (also called pits or active sites) using its pit detection algorithm. With crystal structures for E-pro, M-pro complexes, and S-gly, the program generally identified different binding sites where the lowest score value was considered the best result. Of these five predicted cavities, the one with the largest volume was selected for consideration, as it includes the conformation of the binder. In each fitting run, the best poses were selected based on their MVD reclassification scores and the average of the 30 reclassification scores was then calculated as the final score for each compound. The MVD score and the best poses reclassification scores for each of the docking studies of the ligands with the macrostructures are summarized in Table 1.

**Figure 3:**
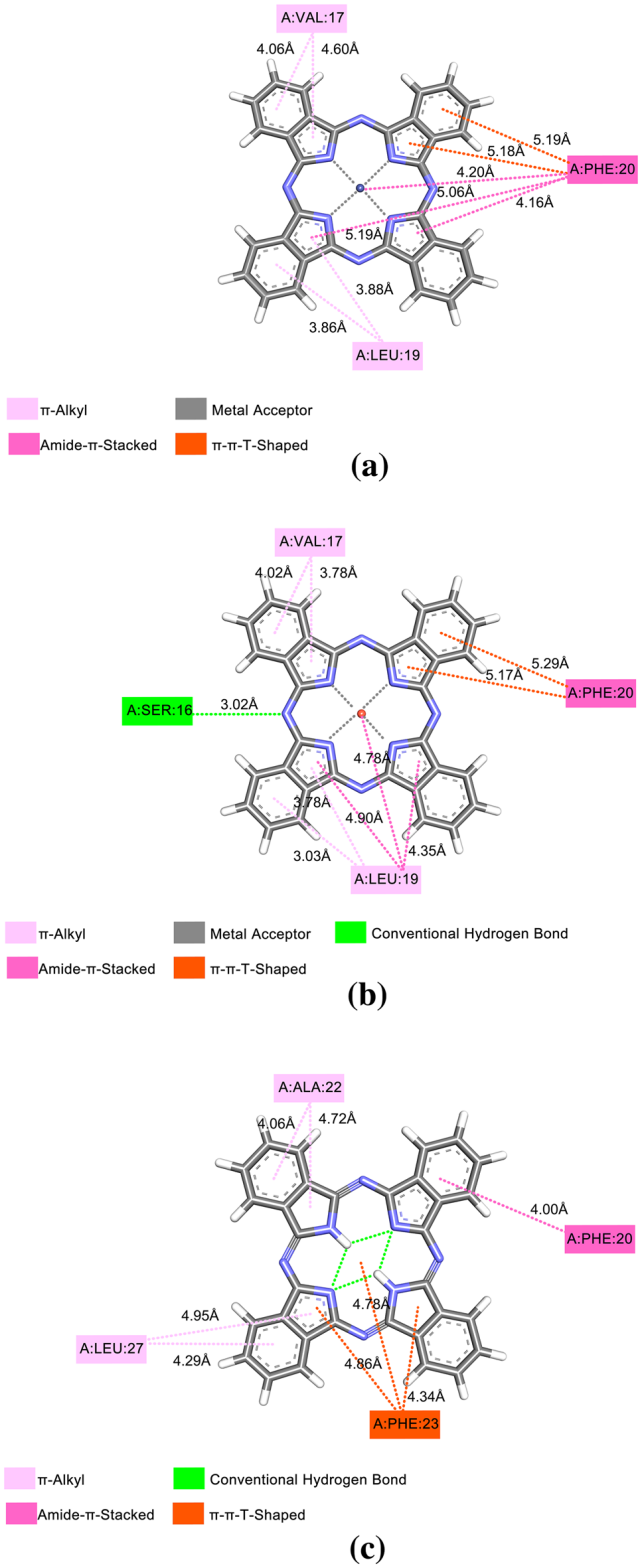
E-pro with: (**a**) CoPc, (**b**) CuPc, e (**c**) NoPc.

Figure 3 shows that molecular anchoring with E-pro, interactions were restricted to amino acids. The interaction of E-pro with CoPc is a total of nine interactions, being four interactions of the π-Alkyl type where there is an interaction of the electron cloud on an aromatic group and an electron group of the Alkyl group generating bonds of the type hydrophobic. Besides three interactions of the Amide-π-Stacked type and three of the π-π-T-Shaped type, which is an interaction between the aromatic ring of LEU:19, it was observed that this interaction is important in the organization of molecules and ligand couplings, such as protein folding and molecular recognition. A detailed description of the π and hydrogen bonds agrees with the coupling analysis, it was found that there is bond affinity, which may indicate a degree of influence of this type of interaction for the affinity energy. Figure 3b shows the interaction of CuPc with E-pro, besides the interactions Amide-π-Stacked, π-alkyl and π-π-T-Shaped formed a conventional hydrogen bond with SER:16, this takes part in the formation of different chemical bonds (such as van der Waals strength, conventional hydrogen bonds, and carbon-hydrogen bonds) with CuPc, in addition to the metal interaction present in CuPc with E-pro. The interaction with NoPc obtained four π -Alkyl type interactions and three π-π-T-Shaped type interactions, non-covalent molecular interaction between the face of an electron-rich π system, the binding energies are significant, with the values of the solution phase falling in the same order of magnitude as the connections. Similar to these other non-covalent bonds, cation-π interactions play an important role in nature, particularly in protein structure, molecular recognition, and enzymatic catalysis. The central part of this ligand obtained, like the previous ones, intramolecular interactions such connections exhibited more favorable energy values. This is because these groups adapt better to the active site of the protein, facilitating the interaction with the amino acids present.

**Figure 4:**
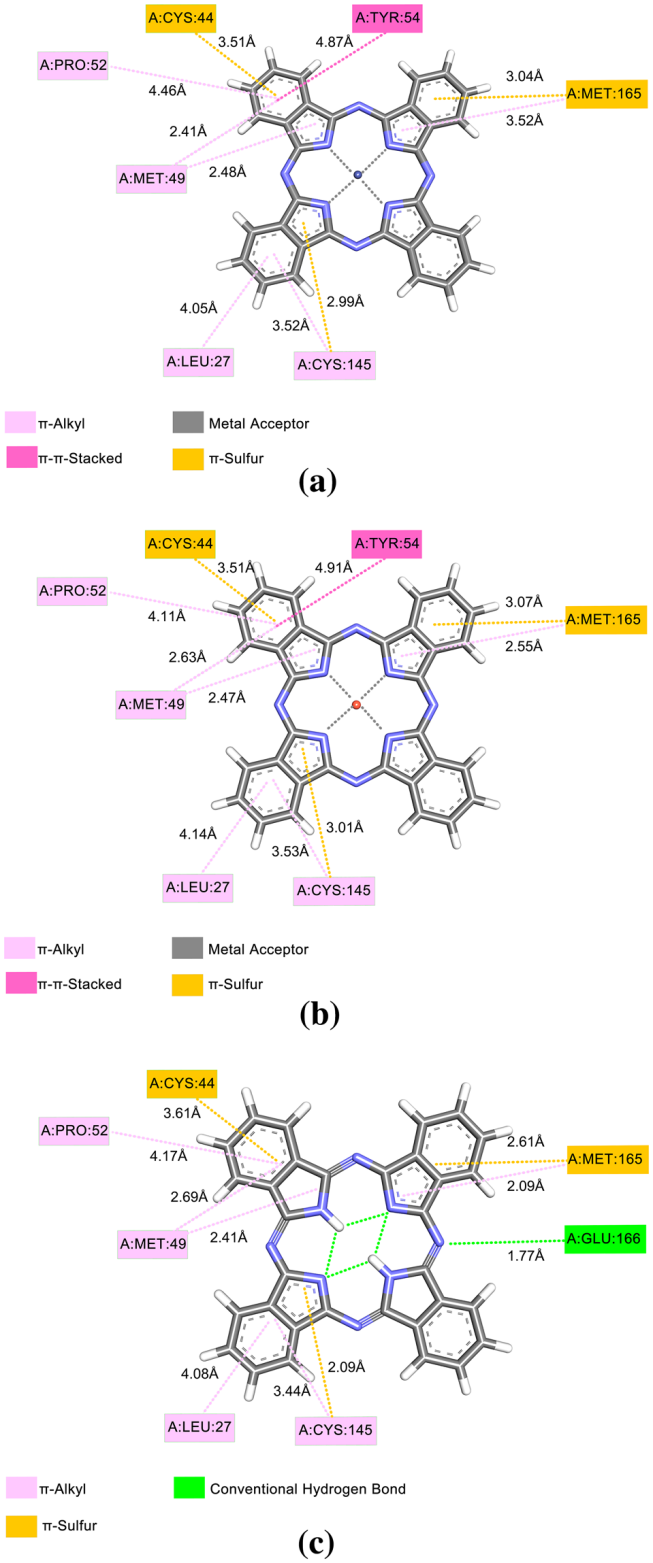
M-pro with: (**a**) CoPc, (**b**) CuPc, e (**c**) NoPc.

Figure 4 demonstrates the interactions of Pc's with M-pro, such interactions occurred in the catalytic site of the protease where amino acids MET49, LEU27, CYS145, CYS44, TYR54, and MET166 are located. Whenever we do protein–ligand docking, what we look at is the conformation of the ligand with which it is binding to the receptor protein, and we try to quantify that binding energy between them using various force field equations. Now, whenever the ligand interacts with the protein, at the atomic level, it is the electrons that are involved in forming covalent or non-covalent bonds. This π-Sulfur, π-alkyl, and π-π-T-Shaped interactions come in the broad category of noncovalent interactions. In pi-π-alkyl interactions, there is an electron cloud interaction on an aromatic group and an electron group of an alkyl group. In the π-π-T-Shaped interaction there is an electron cloud interaction between two aromatic groups, but in a T shape, side electron cloud of a ring and electron cloud of another ring. In the π-Sulfur interaction, the pi-electron cloud of the aromatic ring interacts with the lone pair of electron clouds of the sulfur atom.

**Figure 5:**
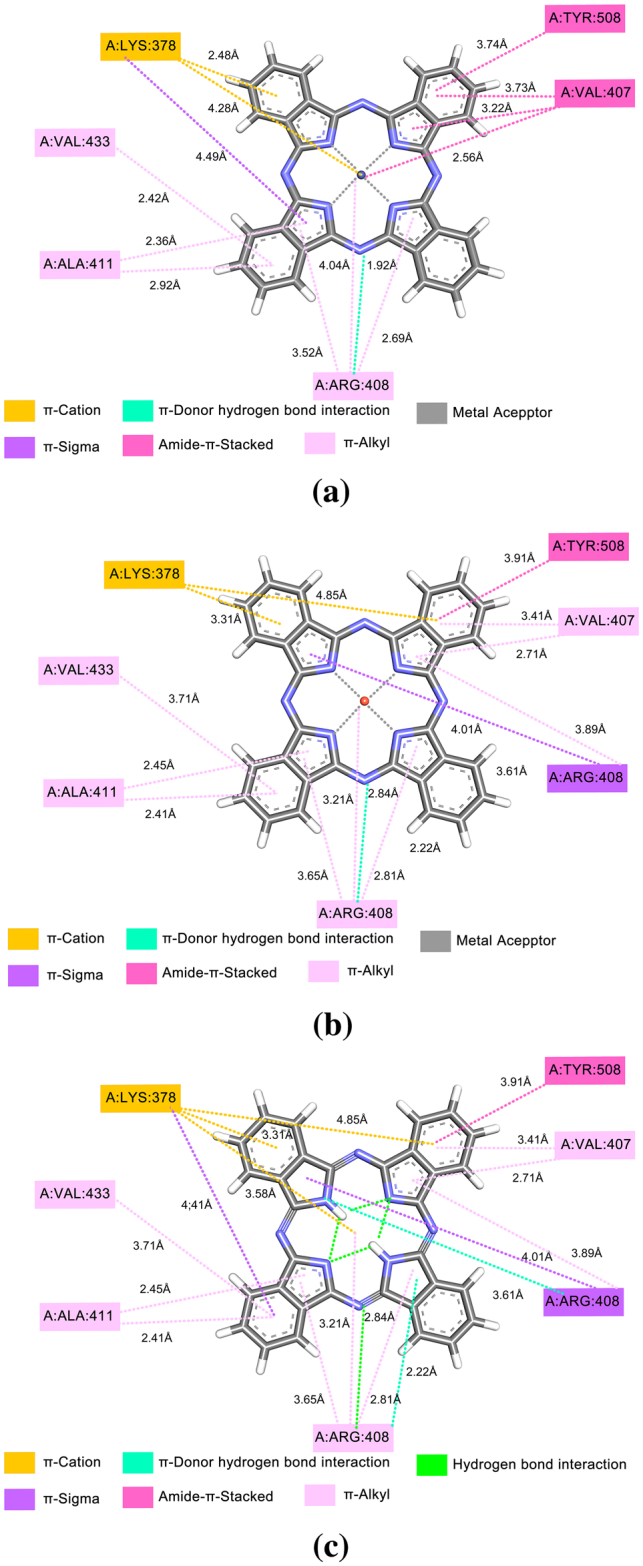
S-gly with: (**a**) CoPc, (**b**) CuPc, e (**c**) NoPc.

Due to the crucial role of S-gly in the SARS-CoV-2 infection process, this structural component may represent a target for neutralization mediated by antibodies or small molecules. Essential atomic-level information to guide the design and development of inhibitory agents. The essential amino acids from the S-gly active site were compared with those reported before the docking study to validate the selection of the correct binding pocket. Figure 5 shows the interactions that the ligands had on glycoprotein S with CoPc, the glycoprotein had interactions of the types π-alkyl, π-cation, π-sigma, amide-π-stacked, π-hydrogen donor and metal acceptor being the latter intramolecular. From the interaction between the receptor and the ligands, considering such interactions, the DOC results revealed interactions with the so-called active site Glycoprotein, where there is a greater chance of binding attached to the molecular targets in question. Analyzing the distances of the interatomic interactions and from the DOC results, it is observed that the interaction mode predicted by the positions may indicate that it has a high capacity for interaction. NoPc had the highest amount of bonds such as hydrogen, the main molecular bond interactions, and the calculated affinity energy was used to assess the reliability of the predicted complex, DOC could identify a promising conformation. Another important aspect to note is that the S-gly catalytic site has hydrophobic characteristics. A detailed description of the π and hydrogen bonds agrees with the coupling analysis, it was verified that there is bond affinity, which may indicate a degree of influence of this type of interaction for the affinity energy. The formation of hydrogen bonds in almost all interactions except S-gly was observed in all ligands, while London forces are formed with aliphatic groups. As the bonds are very close and have attractive and hydrophobic characteristics, they can therefore relate that such interactions with macromolecular structures contributed to the formation of better associations, exploring the electronegativity differences between atoms. The positions assumed by the ligands in the active site allow interactions with the amino acids present. Each position taken can lead to associations with different local amino acids. The better the binding energies, the stronger the interactions that occur between the ligand molecules and the amino acids.

### MESP analysis

**Figure 6:**
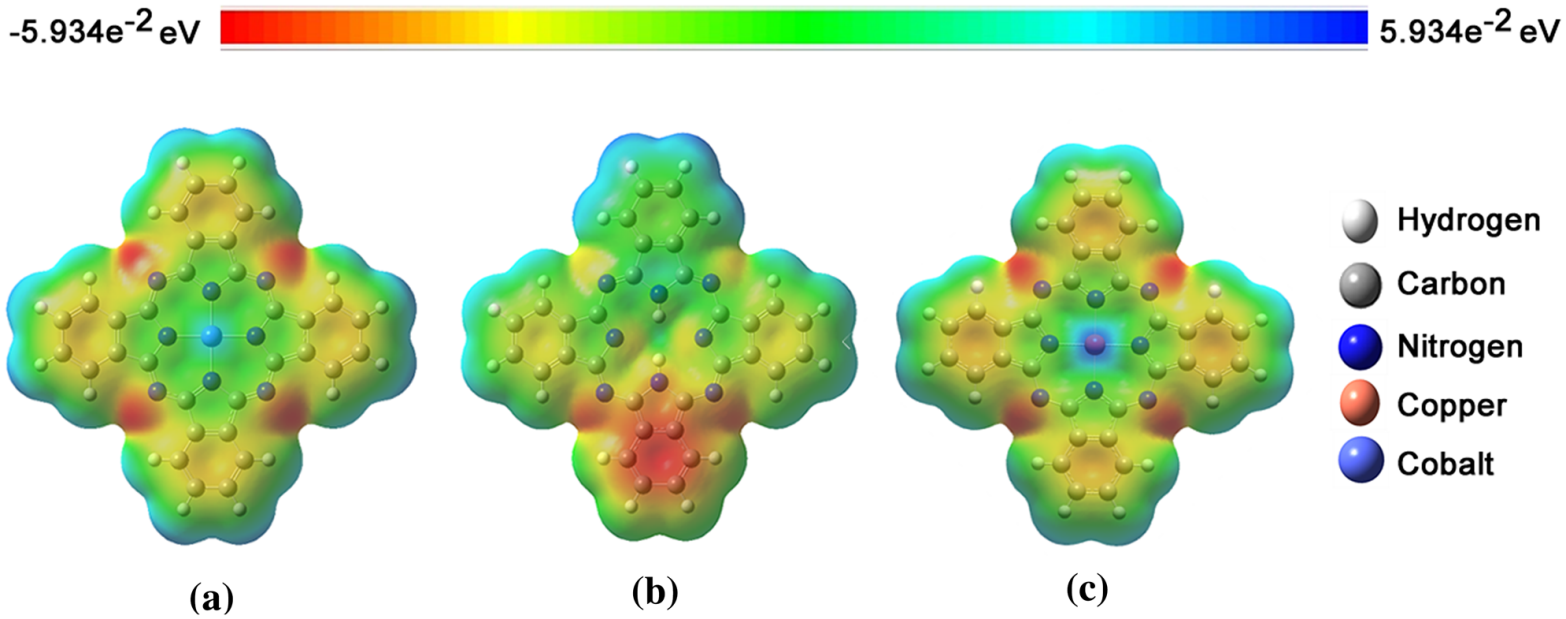
The MESP for: (**a**) CoPc, (**b**) NoPc, e (**c**) CuPc, B3LYP / LANL2DZ level of theory.

MESP plays a key role in the initial phase of bioactive conformation, explaining receptor-ligand interactions. The red, green, and blue colors indicate the high accumulation of negative charge, neutral region, and positively charged region, respectively, as seen in Fig. 6. The negatively charged region of the Pc's and the surrounding groups play a key role in the interaction with the macrostructures so that the electrostatic potentials of the inhibitors influence the inhibition effect. The MESP plotted for Pc showed the most electronegative potential region (red color) in the oxygen atom in the chemical interactions present. The MESP for polar molecules like Pc reveals sites that are richer in electrons and poorer in electrons.

In Fig. 6, it is possible to observe that the molecular contour of NoPc is more electropositive and, therefore, contributes little to the electronic displacement in its molecular structures (Fig. 6b). This property remains after the addition of transition metals to Pc, although the Co and Cu atoms, when inserted into the molecule, also become punctually electropositive regions. On the other hand, it is observed that the addition of transition metals (Co and Cu) generates an alteration in the electronegative regions of the NoPc molecule. The electrons initially arranged in aromatic rings (Fig. 6b), were transferred to the four nitrogen atoms, symmetrically positioned in the molecular structure (Fig. 6a and c). Therefore, it is verified that transition metals invert the electrical charge signal of these nitrogen atoms, generating a quadrupole in the molecular geometries of CoPc and CuPc.

However, true polar molecule MESPs generally do an excellent job predicting the possibility of charge-dipole and dipole–dipole interactions. The MESP is widely used as a reactivity map showing the regions most likely for electrophilic attack by reagents, similar to charged points on organic molecules, as well as providing a simple way to predict how different geometries might interact. The complex's MESP is obtained based on the result optimized with the B3LYP / LANL2DZ base. CoPc and CuPc have five possible sites for the electrolytic attack. The negative regions are partial to carbon–carbon double bonds within the ring and mainly over the region between H, C, O, and the metals; while NoPc presents variations of electronegativity on its surface. As we mentioned earlier, electrostatic potential is mainly used to predict relative locations and reactivity to electrolytic attack and in studies of biological recognition and hydrogen bond interactions. In all cases, the magnitudes of the MESP close to the oxygen atom of the C2-OH group (O2) are highly increased. Compared to magnitude in other areas.

**Figure 7:**
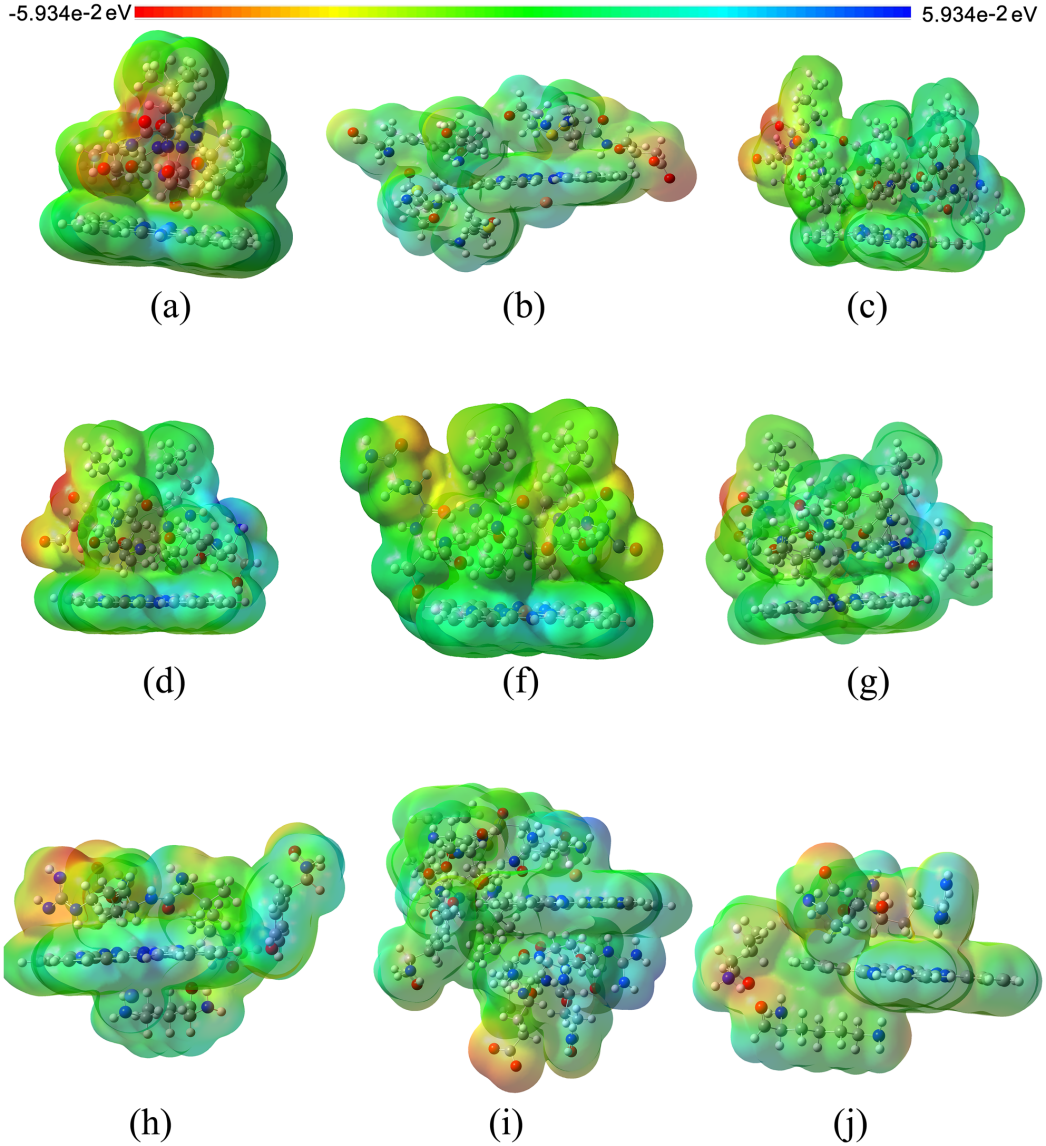
The MESP for E-pro: (**a**) CoPc, (**b**) CuPc, and (**c**) NoPc; M-pro: (**e**) CoPc, (**f**) CuPc. and (**g**) NoPc; and S-gly: (**h**) CoPc, (**i**) CuPc, and (**j**) NoPc), with B3LYP / LANL2DZ level of theory.

The MESP is a very useful descriptor in understanding sites for electrophilic attack and nucleophilic reactions and for studying the biological recognition process. Figure 7 provides a visual presentation of chemically active sites and comparative reactivity of metal atoms when phthalocyanines interact with active sites of each proposed macrostructure. The potential has been very useful as an indicator of the sites or regions of the receptor that are initially attracted by the electrophile/nucleophile approximation, and it has also been successfully applied to determine the best relative orientation of each ligand. The electrostatic potential value is largely responsible for binding a substrate to its receptor-binding sites since the receptor and corresponding ligand recognize each other on their molecular surface. In the present study, we consider the interactions of Pc with amino acids close to certain locations in a series of molecules obtained using continuous electron density and are well known to be reliable measures of their relative hydrogen bond acceptance strengths.

MESP techniques have been used as independent strategies in the study of active compounds and lead to the proposal of new molecules for synthesis and biological tests. The joint applications of these powerful tools were carefully described to unravel the structure–activity relationship of bioactive compounds, consequently proposing new molecules. In Fig. 7a we observe that in the interactions of E-pro with CoPc and NoPc there are regions of electrophilic and nucleophilic reactivity trend, while in the interaction with CuPc it shows only an electrophilic reactivity trend, and it is also possible to see that the presence of Co influences the increase in charge density on the E-pro. The MESP was sensitive to small atomic distortions and it was found that the orientation of the interlayer water molecules is influenced by changes in it. The MESP was analyzed in the range of − 5.934e−2 eV to 5.934e−2 eV which indicates that the surface is mainly electrophilic in nature. Figure 7b shows the MESP map of the Pc cluster with the M-Pro where the negative charge is offset by a proton present at the oxygen bridge site. Although the overall topography of the MESP maps is similar, there are still some small but subtle changes. It can be verified through the electrostatic potential calculations obtained by the B3LYP/LAN2DZ method, that the pair of unbound electrons of the nitrogen atom is present. In Fig. 7c we observe that in the interactions of S-gly with CoPc, CuPc, and NoPc there are regions of electrophilic and nucleophilic reactivity trend where it is possible to see that the presence of Co possibly influences the increase in charge density in E-pro relating to the results obtained in the MoldockScore, as seen above in Table 1. Through the results in Table 1, we can verify that the lower the value of the MoldockScore, the ligand + receptor has a greater affinity, thus explaining the concentration of charges in E-pro being higher when interacting with CoPc and lower with NoPc. The higher concentration of charges makes the receiver with a greater tendency to reactivity for possible connections. This result shows that inhibitor molecules must not always have essentially the same chemical groups like those found in the original substrate present in the catalytic site, which, in the case of protein kinases, favors the evolution of cellular signals. The corroboration of these interactions for the studied inhibitors was not always achieved through the measurement of hydrogen bonding distances. However, the results obtained via these approximations should be corroborated with more advanced calculations using the LANL2DZ density functional consonant with B3LYP that take into account more electronic variables for a better description of the molecular complex.

### Molecular dynamic analysis

#### RMSD

**Figure 8:**
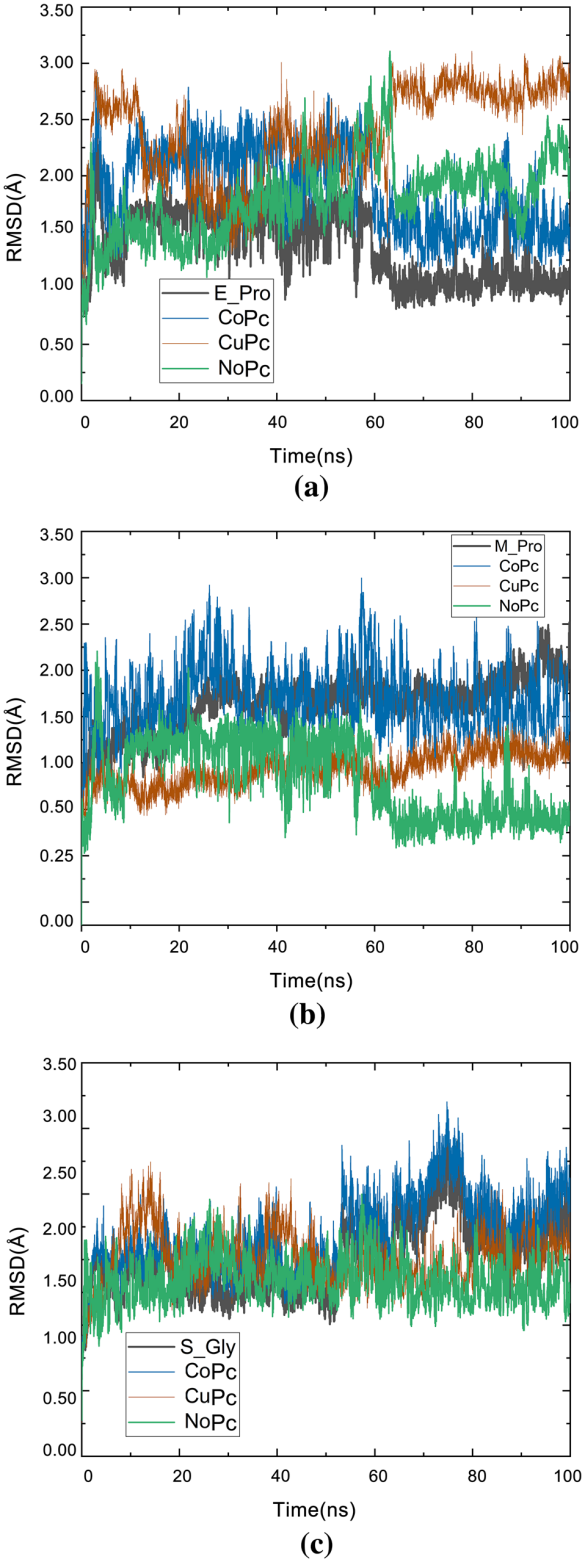
RMSD versus time of ligands with (**a**) E-pro; (**b**) M-pro e (**c**) S-gly.

RMSD measures the average distance between atoms of superimposed structures, thus being commonly used for similarity comparison. Furthermore, the RMSD values ​​can also provide information about the system's equilibrium, that is, the moment in which the structure converges on its most stable mean conformation. At the beginning of the simulation, the values ​​tend to increase sharply while the structures try to balance themselves until they reach a plateau that suggests that the structures have reached equilibrium. As RMSD values ​​are given as a function of time, this analysis allows observation of the period that structures take to stabilize. Here, the difference was measured between the atoms of the starting backbone structure (the crystallographic model) and each structure obtained during the subsequent frames of the simulation trajectory. To validate and confirm the stability of the suggested protein–ligand complexes, we performed MD simulation at 100 ns for the three Pc with each SARS-CoV-2 surface protein structure identified in our DOC studies. The RMSD for each complex was calculated (Fig. 8). The RMSD value can predict the stability of the ligand complex of MD runs. A lower RMSD value indicates greater stability of the protein complex. We calculate the RMSD of the complexes concerning the Cα atom concerning the MD simulation time. Overall, the mean RMSD for all complexes was low, ranging from 1.12 to 3.33 Å. Thus, the results of RMSD analyzes of the trajectory for structures in complex with E-pro were compared to those of the protein to note the behavioral differences in the balance and stability of the structures, as can be seen in Fig. 8a. It was observed for E-pro that the RMSD values ​​increased during the trajectory, mainly for CuPc and NoPc. Simulation with CoPc obtained more stable RMSD values ​​compared to E-pro. From Fig. 8a, it can be seen that the CoPc and CuPc molecules have more stable interactions, with lower RMSD values compared to the NoPc molecule. This result is in agreement with the results predicted in the docking simulations, discussed previously in item 3.1, since after the addition of the transition metals, the Pc molecule started to present stronger bonds with E-pro.

The RMSD graphs were calculated taking as reference structures corresponding to the 0 ns times of simulation. In Fig. 8b, the curves for CoPc are shown following the path of the protein, which predicts that there was a smaller-distance interaction between receptor-ligand. In comparison with the RMSD curves of the CuPc and NoPc fragments, it is observed that the curves of these fragments reach a higher deviation level, but are still close to the M-pro. Figure 8b compares the RMSD curves of the three Pc's understudy. In general, S-gly remained more stable in the presence of bound Pc when compared to its initial conformation. As shown in Fig. 8c, the RMSD of all three complexes reached equilibrium slightly increased around 70 to 80 ns and then gradually stabilized towards the end of the beginning of the MD simulation. After about 100 ns, the RMSD values ​​converged between 1.0 and 2.5 Å. The stability of the initial RMSD in the complexes was expected due to the interaction of the inhibitor with the protein, which decreased the overall flexibility of the protein. This is possible because of the better interaction profile of the compound with the catalytic site. However, a more detailed analysis of the protein skeleton flexibility as possible from the greater range of motion that occurred because of a decrease in flexibility in the S-gly binding region, which revealed the influence of glycoprotein interactions on ligands. The results below also imply that the OPLS-AA force field and CHARMM36 force field (under the tip3p water model) accurately describe the structure of the Pc + Receptor complexes. Thus, S-gly was the protein that had the most stable interactions with Pc's. This result is also in agreement with what was previously observed in the results of the docking simulations, as S-gly was the protein that presented more interactions with the Pc's. Consequently, during MD simulation these molecules showed to have more stable conformations.

The interactions of metallic chemical bonds in CoPc and CuPc show that the electrostatic interactions of a polar group with its surroundings can be described by a simple model of a dipole with constant momentum under the action of a force field. This relationship is used to develop a general approach to generate a charge model based on electrostatic energy for molecules containing polar chemical bonds. The MD simulations of the ions present in the Pc's with the active site of the external structures of SARS-CoV-2 provide a better representation of the electrostatic interaction in the bonding environment, the CHARM36 force field simulations suggest the charges can also be related to changing the receiver in simulation.

#### SASA

**Figure 9:**
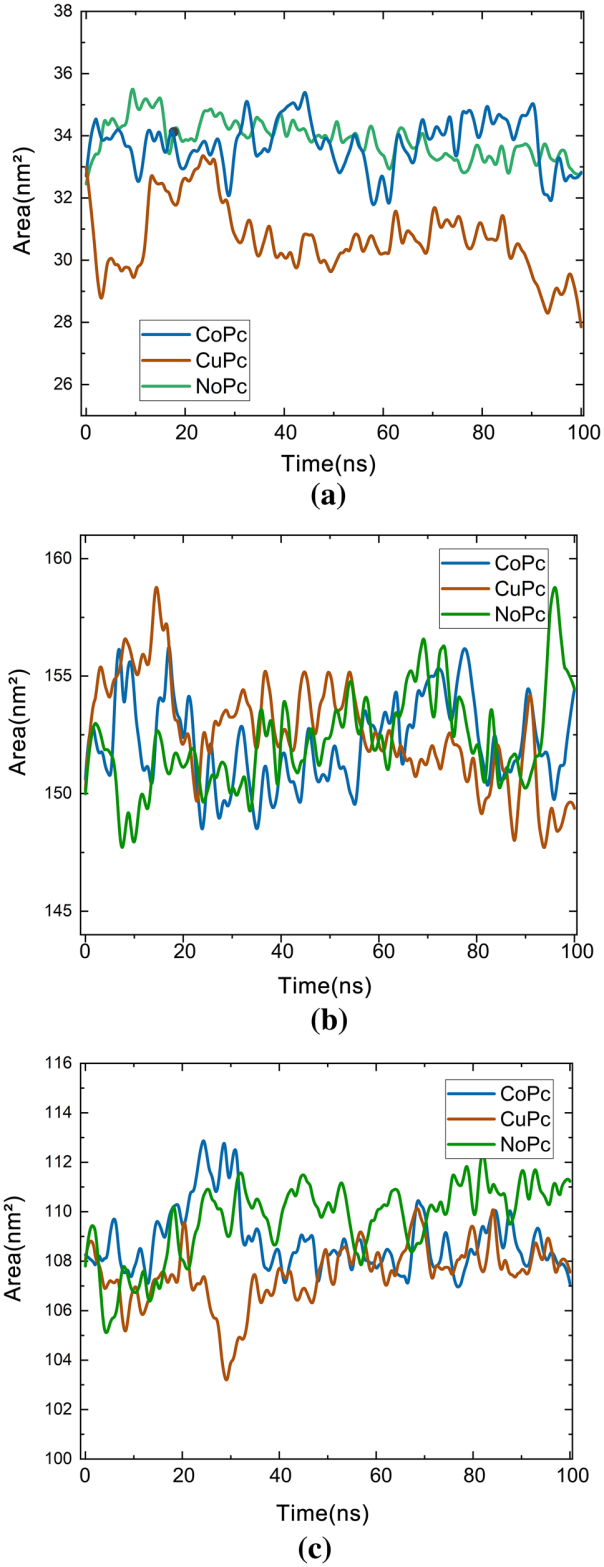
SASA results: (**a**) E-pro (**b**) M-pro, e (**c**) S-gly.

SASA was calculated for three complex systems to measure the interaction between protein–ligand complexes and solvents using the g_SAS module in the GROMACS package. Polar and non-polar surface areas are often defined using partial atomic charges taken from the molecular potential used. These partial atomic charges differ significantly between force fields. To avoid this force field dependence, we recalculate the surface areas accessible to average polar and nonpolar solvents, adding the contributions not according to the partial charges of the atoms, but according to whether these atoms belong to hydrophobic or hydrophilic residues. This can be done using the option for residuals in the g_SAS tool. The solvent-accessible surfaces of the terminal amino acids are much larger and are not dependent on force fields, indicating that they are extensively exposed to the solvent (Fig. 9). The central residues that have the smallest mean square fluctuation are also the residues that have the smallest area exposed to the solvent, as they are in the most central part of the receptor structure and, therefore, protected from the solvent. The apolar solvation free energy was estimated using the SASA. The free energy of the non-polar solvation of each atom in a molecule is proportional to the SASA. The non-polar term is responsible for the rearrangement of solvent molecules around the solute and the van der Waals (vdW) contact interaction between the solute and the solvent molecules. E-pro conformational changes modeled over the simulation period were estimated using SASA calculations. The average SASA value calculated for E-pro for CuPc and NoPc during the 100 ns simulation was relatively stable, showing that there were no significant changes in the E-pro structure, but in the interaction with CoPc, there were significant variations in the period in the period. from 10 to 30 ns as seen in Fig. 9.

The results confirmed that the M-pro residues were well exposed and accessible to the solvent. In Fig. 9, SASA shows the surface area of ​​the receptors that is accessible to a solvent. The SASA range of this protein structure for M-pro interaction is between 15 to 20 ns and 60 to 80 ns, where the receptor surface becomes more accessible. We can see the lowest SASA value in Fig. 8c for the CoPc for the time 20 to 40 ns at the start of the simulation period, for the other two interactions, the SASA value of this protein structure is considered constant. We can see from all the graphs in Fig. 8 that increasing the SASA value showed a decrease in the amount of protein, which indicates that it shows a decrease in the solvent-accessible surface area, which increases protein stability. From the graph, we can see that the SASA value of the structure formed by receptor + CoPc is globally higher when compared to the other interactions, making this interaction more stable. Relative SASA can predict protein conformational changes after ligand binding. According to the SASA results; it was observed that the binding of Pc induced small conformational changes in the viral structures.

#### Free Energy

The dynamic behavior of selected compounds is analyzed for low energy profiles using the G_MM/PBSA script, which uses the MM/PBSA method, which is used for post-processing of coupled structures along with the reliability of the compound binding within the pocket of Flexible connection. The 100 ns simulation of protein–ligand complexes together with the free energy of binding of MM-PBSA suggests that the main molecules fit perfectly into the binding site and are structurally stable with a low energy profile. The MM-GBSA method was calculated by the free energy (ΔG_Binding_) of the Pc with the viral structures, and this was done using the surface area energy, solvation energy, and energy minimization of the ligand and receptor complexes. The analysis of vdW energy variation for this interaction aimed to investigate the structural properties of Pc. Thus, vdW interactions play an important role in the properties of systems in which much stronger dipole–dipole interactions are present. The first part of the potential energy of the solvent includes linked terms like angle and torsional energies as well as unlinked terms like vdW and electrostatic interactions. The second term is responsible for the dissolution of different species. It is quantified by the sum of two energy terms, the polar and non-polar solvation energies using an implicit solvation model.

The values ​​for SASA energy in Table 2 show a marked increase as the interaction approaches the surface of SARS-CoV-2, indicating structural relaxation. Thus, we assume that simulation times 100 ns were sufficient to sample balanced systems. The highest SASA energy value is found for the interaction of CoPc with S-gly. Several alternative non-polar solvation models, along with the widely used SASA model, are also included. Furthermore, the binding energy can be decomposed by residue. In several recent investigations, the non-polar solvation energy obtained using the widely used SASA model correlates poorly with those obtained in the explicit solvation simulations. Several other models, however, were developed. The parameters for these models were optimized based on explicit solvent simulations and validated against free solvation energies. The G_MMPBSA provides options to use three non—polar alternative models. The influence of model choice on binding energy was therefore examined. Non-polar values ​​were calculated using these different non-polar models with the parameters shown in Table 2.

**Table 2:** Energy components.

Receptor	Pc	Van der Waals (kJ/mol)	Electrostatic (kJ/mol)	Polar solvation (kJ/mol)	SASA Energy (kJ/mol)	ΔG_Binding_ (kJ/mol)
E	CoPc	− 124.289 ± 3.98%	− 6.502 ± 8.723%	72.304 ± 3.026%	− 18.056 ± 3.172%	− 92.730 ± 8.876%
	CuPc	− 100.370 ± 4.011%	− 4.689 ± 7.985%	68.806 ± 3.099%	− 11.776 ± 1.239%	− 83.842 ± 7.157%
	NoPc	− 94.806 ± 2.968%	− 3.576 ± 9.423%	64.655 ± 5.248%	− 9.045 ± 2.858%	− 49.772 ± 8.434%
M	CoPc	− 157.004 ± 5.387%	− 9.564 ± 7.584%	103.025 ± 4.025%	− 17.078 ± 3.027%	− 102.568 ± 6.998%
	CuPc	− 121.368 ± 5.069%	− 6.278 ± 6.141%	96.837 ± 3.532%	− 12.776 ± 6.613%	− 87.842 ± 6.838%
	NoPc	− 100.370 ± 4.563%	− 4.502 ± 9.485%	68.806 ± 5.067%	− 11.776 ± 5.681%	− 64.842 ± 7.837%
S	CoPc	− 179.720 ± 3.884%	− 12.442 ± 6.747%	125.747 ± 2.998%	− 32.584 ± 2.832%	− 182.443 ± 3.799%
	CuPc	− 133.370 ± 0.768%	− 9.502 ± 4.167%	91.806 ± 4.639%	− 21.776 ± 8.163%	− 158.954 ± 7.968%
	NoPc	− 121.370 ± 9.288%	− 7.502 ± 9.485%	85.806 ± 7.231%	− 18.126 ± 4.665%	− 129.963 ± 4.967%

The electrostatic energy analysis of the Pc interaction significantly increased as structures plus the surface of the virus were tested, which may contribute to the observed redshift of the emission maxima. The presence of ions in the system may have caused a greater change in their ground state electrostatic energies. The lack of changes in binding affinities indicates that free energy landscape sampling using MMPBSA is largely affected by the observed domain movements, in all cases the values ​​present in the electrostatic terms offset each other, resulting in minimal changes in structure. Electrostatic attraction is considered a common feature of all ionic systems, using both simulation strategies. The balance of these two effects is predominantly responsible for the G_MMPBSA obtained for each system. The general electrostatic energy of Pc with 2 + ions present in its structure is almost independent of the type or concentrations of ions. The results of the energy analysis of the complexes provided in Table 2 demonstrate that the ΔG_Binding_ was − 92,730 kcal/mol (CoPc), − 83,842 kcal/mol (CuPc), and − 49,772 kcal/mol (NoPc), for E-pro. The ΔG_Binding_ values ​​were − 102,568 kcal/mol (CoPc), − 87,842 kcal/mol (CuPc) and − 64,842 kcal/mol (NoPc), for M-pro. For the S-gly the results obtained values ​​of − 182,443 kcal/mol (CoPc) − 158,954 kcal/mol (CuPc) − 129.963 kcal/mol (NoPc). The results show that the contributions to the ligand coupling were the polar solvation terms, SASA, and vdW. These identified inhibitors do not represent any major changes in their free binding energies. Details of the MM-PBSA calculation of the complexes are summarized in Table 2. The Van der Waals potential energy (E_vdW_) varies mainly by two factors, specifically, surface area (molecule geometry) and electronic polarizability (molecular size), among the different types of calculated energy, the ones that gave the highest percentage error were those for the NoPc, this may be due to the absence of the metal in its environment. We can relate this to the CoPc and CuPc results, which show that when there is the presence of the metal, the chances of more vdW and ΔG_Binding_ interactions are higher, and their percentage error is smaller. Thus, it can be suggested that S-gly is the protein with the greatest capacity to interact with Pc's, since its binding free energy was the most expressive compared to the other proteins investigated in this paper. Furthermore, as the interactions gradually increase with the addition of transition metals, so that the Protein + Ligand complex composed of S-gly and CoPc were the most interacting, it is observed that these results have an excellent correlation with the data from RMSD and docking simulations.

Large molecules are generally associated with greater polarizability. Pc's with metal are more polarizable as there are more electrons to deform and interact. Electrostatic potential energy is influenced by the polarity of interacted molecules that can be expressed by a dipole moment. The greater the difference in the electronegativity values ​​of the bonded atoms, the greater the dipole moment. The existence of central heteroatoms plays a significant role in increasing the molecule's polarity. Among the interactions, those with S-gly stand out, ΔG_Binding_ values have a strong affinity value, demonstrating the effectiveness of the interaction with glycoprotein residues. The E-pro also shows a strong affinity value, the ligands do not significantly interact with the binding site residues and it also has a high instability, which translates into a high standard deviation value, the interaction with M-pro remains with intermediate value, even so, it demonstrates stability when compared to E-pro, this last receptor is not so suitable when it comes to interaction for the inhibition of SARS-CoV-2, but the interaction within the binding site persists throughout the simulation.

## Conclusion

This study reports potential inhibitors for the major surface structures of SARS-CoV-2, through an integrated computational approach for repositioning inhibitor agents. After our fit tests, a divergent binding posture was generated, and the posture with the ideal fit score and interactions was considered to be the best posture for further processing and analysis. The DOC of the compounds for the surface structures was visualized in terms of interactions in the protein substrate recognition pockets, and the dynamic stability of the receptor-ligand contacts was evaluated using MD simulations of each type of Pc. Molecular anchoring with multiple protein conformations followed allowed adjustments in the receptor conformation through the DOC approach. Using RMSD, how ligands interact over time, and how this interaction occurs, since Pc was able to bind residues from the active site of macromolecules, they showed good interactions. Based on MD simulation studies, it demonstrated close values ​​for the protein so that the pose of the ligand was considered to be the most stable for all interactions. Thus, we believe that obtaining information about the molecular mechanism responsible for the recognition of protein–ligand through this study will facilitate the development of equipment to combat the disease COVID-19. Simulations can also explain surface functionalization and its impact on protein interactions. Through the post-processing of the trajectory, it was possible to identify the main driving forces of the adsorption (hydrophobic effects, hydrogen bonds and calculate the interaction energies to obtain a quantitative estimate of the binding energies.

The binding mode of ligands was known to understand the binding properties and the mechanism of action of the interactions. From the combined results of coupling and free energy calculations, it was found that residues from the active site of S-gly interacted strongly with Pc. Prediction of the glycoprotein binding site could help in the discovery and design of different new potent agonists. The work reported here addresses an important concern and urgent need for medications to treat SARS-CoV-2 infection. As shown through this integrated approach, computational prediction for the inhibition of the main external structures of SARS-CoV-2 has resulted in some promising leads for further experimental validation. Overall, our computational PC repositioning strategy predicts some promising drug candidates that, if confirmed through experimental and clinical approaches, could contribute to solving the global crisis of the COVID-19 pandemic. In addition, MD simulation was used to understand the conformational changes in the binding protein complexes. In the analysis of MM-GBSA, molecular docking studies were validated and it was shown that the ligands have interaction characteristics capable of adsorbing proteins.

## Data Availability

Data used to support this study are included in the article.
